# Allogeneic materials in complications associated with pre-implantation restoration of maxillary and mandibular alveolar processes. A four case report

**DOI:** 10.1007/s10561-013-9398-5

**Published:** 2013-09-27

**Authors:** Marta Krasny, Kornel Krasny, Artur Kamiński, Piotr Fiedor

**Affiliations:** 1 Department of Orthodontics, Warsaw Medical University, Warsaw, Poland; 2 Medicare Dental Practice, Warsaw, Poland; 3 Department of Transplantology and Central Tissue Bank, Warsaw Medical University, Warsaw, Poland; 4 Department of General and Transplantation Surgery, Transplantation Institute, Warsaw Medical University, Warsaw, Poland

**Keywords:** Allogeneic granulate, Allogeneic block, Augmentation complications, Bone replacement material

## Abstract

There are numerous types of bone replacement materials used to regenerate atrophic alveolar processes before the elective intraosseous implantation. Properties of these materials differ one from another, therefore the choice of material should be thoroughly analysed as well as its type and texture in regard of intraoral conditions and the objective to be achieved. The study involved reconstruction of atrophic alveolar processes with allogeneic bone following unsuccessful use of synthetic and animal materials. The procedure of bone regeneration was performed with frozen bone block (case 1) and allogeneic bone granulate (cases 2, 3, 4) radiation-sterilised with 35 kGy prepared by the Tissue Bank. In all of the presented cases after 3-month implant reorganisation optimal width of the process was obtained, which allowed implant embedment (case 1) or correct implant submergence in the osseous tissue, when implantation took place at the same time (case 2, 3, 4). Allogeneic bone material both, in the form of a block as well as granulate, seems to be an adequate alternative for other materials used in order to widen the bone of the alveolar process, particularly in difficult, complicated cases, where the first regeneration procedure was not successful.

## Introduction

Correct, aesthetic and long-lasting prosthetic implant-supported restoration often requires filling of bone defects in the alveolar process with some bone substitute. Dental market offers a range of xenoplastic and alloplastic materials used for this purpose. The former type is of animal origin (bovine bone is used most commonly) and includes Deproteinised Bovine Bone (DBB), which constitutes a scaffold resembling human bone with osteoconductive properties as well as Demineralised Bovine Bone Matrix (DBBM). DBBM shows osteoinductive properties owing to the presence of Bone Morphogenic Proteins (BMPs) (Bauer and Muschler 2000). The products are available in granulate or block form.

Whereas, alloplastic materials are manufactured synthetically, may be of natural, non-organic origin (e.g. hydroxyapatite or bioactive glass) or organic origin (e.g. algae or coral). Their use is burdened with the risk of prion infection or spreading other pathogenic factors. The materials provide only a scaffold for the future bone; hence they do not show any osteoinductive properties (Bauer and Muschler 2000).

Alternatives of the abovementioned materials include non-cellular (biostatic) allogeneic products (Holmquist et al. 2008), in the form of demineralised bone-lyophilised FDBA (Freeze-Dried Bone Allograft), frozen materials or Demineralized Bone Matrix (DBM). When the demineralised bone material is prepared, decalcifying substances are used, which leave type I collagen only and non-collagen matrix proteins, e.g.: BMPs, glycoproteins, and proteoglycans (Bauer and Muschler 2000). Apart from osteoconductive potential the process also provides ostoinductive potential (Boyan et al. 2006). In order to guarantee safety of the human formulations of this type, the donors undergo detailed qualification procedures and are examined for HIV, hepatitis virus, and syphilis infections.

In order to provide successful bone grafting and reorganisation, apart from the scaffold for the newly formed bone, revascularisation must be possible, i.e. vessels must penetrate the structure of the material embedded in the recipient’s body. The vessels allow transport of osteogenic cells, growth factors, and nutrients necessary for reorganisation of augmentation material. Properties of the materials differ; therefore, individual products are not recommended to be used interchangeably. They must be accurately selected depending on the intraoral situation (Khan et al. 2005).

Ignorance of the properties of bone-replacement materials as well as the limitations of their use may lead to serious complications of pre-implantation base preparation and, as a consequence, provoke further resorption of the patient’s own bone.

## Objective of the study

The objective of the study was to present efficacy of allogeneic materials in the form of bone granulate and bone blocks in reconstruction of atrophied alveolar processes of the maxilla and mandible in complicated cases following unsuccessful use of other bone-replacement products.

### Case 1

Patient K. O. aged 37, reported to restore missing tooth 11. According to the patient, the upper right incisor was extracted due to pain complaints and inflammation several months before. The alveolus was filled with alloplastic resorbable bone-replacement material to maintain optimal shape of the alveolar process and preserve its volume.

The orthopantomogram performed on the day of examination revealed radiolucent lesion within the bone of the alveolar process in the area of tooth 11. The intraoral examination showed normal mucous membrane covering the process. Nevertheless, the process underwent considerable resorption within the transverse diameter.

Following consultation the patient chose permanent prosthetic restoration based on intraosseous implant. Due to significant bone atrophy the treatment plan involved widening of the atrophic alveolar process. As the defect involved one wall, a bone block from the Tissue Bank was chosen to be used.

**Fig. 1:**
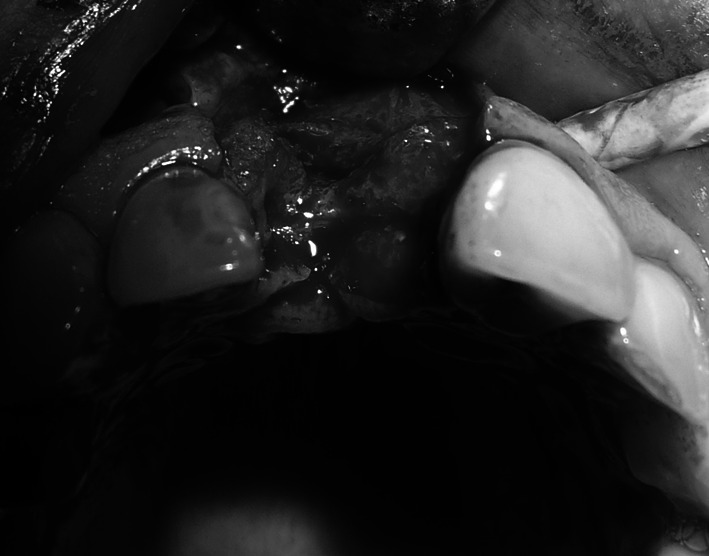
Funnel-shaped defect of the outer bone lamella in the alveolar process

**Fig. 2:**
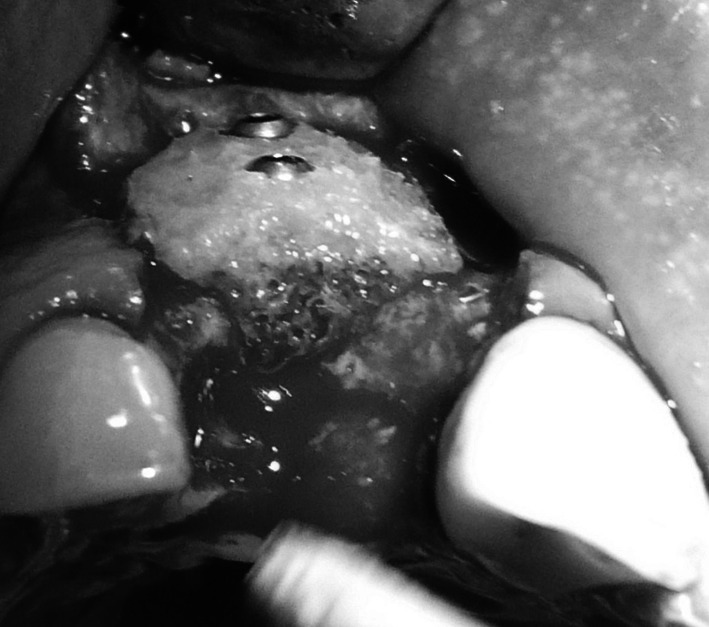
Fixed bone block; view from the vestibule and from the occlusion plane

Under local anaesthesia (Xylonor Forte 4 %) a trapezoidal incision of the mucous membrane was made, preserving interdental papillae. Then the mucoperiosteal flap was separated. Considerable bone defect was found and the measured transverse diameter of the alveolar process was 3 mm only (Fig. 1). The height of the alveolar process did not show significant atrophy. Then, a frozen allogeneic bone block was shaped with drills fixed on a contra-angle handpiece and filled the gutter-shaped bone defect. The fresh frozen bone block composed of compact and spongy bone was prepared at the class C clean room in the Tissue Bank from iliac ala of a deceased donor, defatted and subsequently radiation-sterilised with the dose of 35 kGy in accelerated electron beam. All tissue banking procedures including donor evaluation, laboratory testing, processing conditions, storage in a freezer at −70 °C and distribution were done under Standard Operating Procedures based on national and European legal requirements for tissue and cell banking. The bone lamella of the graft confined the defect from the outside. The adjusted bone block was fixed with 2 Meisinger screws (Fig. 2). For prompter and more certain reorganisation of the graft, both, the block and the adjacent alveolar process were covered with Platelet Rich Fibrin membranes (PRF). After the periosteum was incised, the mucous flap was mobilised and used for covering the surgical site and the wound was sutured.

At follow-up visit 1, which was appointed 2 weeks after the surgery was performed, the sutures were removed. The wound healed normally. The next follow-up visit was appointed 1 month after the grafting procedure. The mucous membrane was found normal with no signs of infection.

**Fig. 3:**
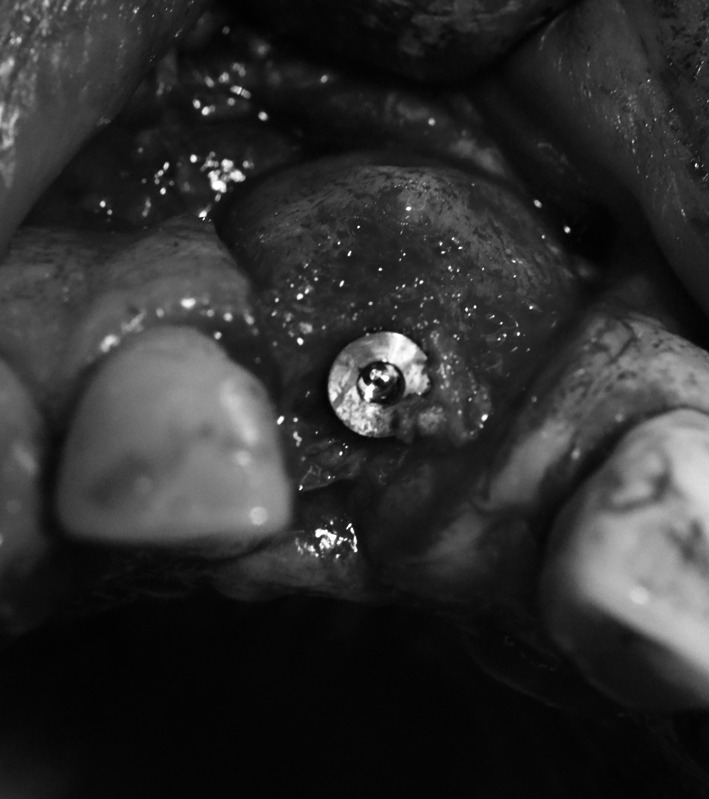
Implant embedded in the bone block, integrated with the process bone

Three months after the procedure a follow-up orthopantomogram revealed a normally healed graft. The findings allowed intraosseous implantation with simultaneous removal of block fixing screws. The intraoral examination revealed normal union and reorganisation of the graft, which bled after drilling. When the implant BIOMET 3I was inserted, primary stabilisation was achieved (Fig. 3), which enabled completion of the procedure and suturing of the wound.

Following 6 months required for normal integration of the screw with bone tissue, porcelain crown was made to restore the missing tooth 11, which completed 10-month dental treatment.

### Case 2

**Fig. 4:**
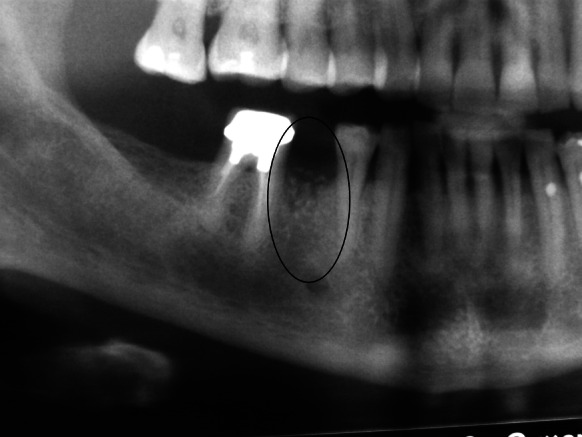
Xenogeneic material, not integrated with the bone within the area of distal root of tooth 46

Patient B. M. aged 42 reported to restore the missing right, mandibular, first molar. According to the patient tooth 46 was extracted 4 months before and at the same time the alveolus was filled with xenogenic bone-replacement material. The orthopantomogram performed at the same visit revealed irregular osteolytic focus of elongated shape resembling the tooth root outline (Fig. 4).

The patient was referred to intraosseous implantation but as X-ray imaging revealed no integration of the material with the bone, removal of the material was planned as well as implant embedment and refilling of the bone defect of the alveolar process with a different bone-replacement material. Due to the funnel-like shape of the defect, granulated bone allograft from the tissue bank was chosen to be used. The fresh frozen non-decalcified bone graft was prepared from deceased donor’s epiphyseal spongy bone. The bone was ground in the LN2 freezer mill, defatted and subsequently radiation-sterilised in accelerated electron beam with a dose of 35 kGy on dry ice. All tissue banking procedures were done under approved Standard Operating Procedures. The ground bone was mixed with the patient’s own bone on 80–20 % basis.

**Fig. 5:**
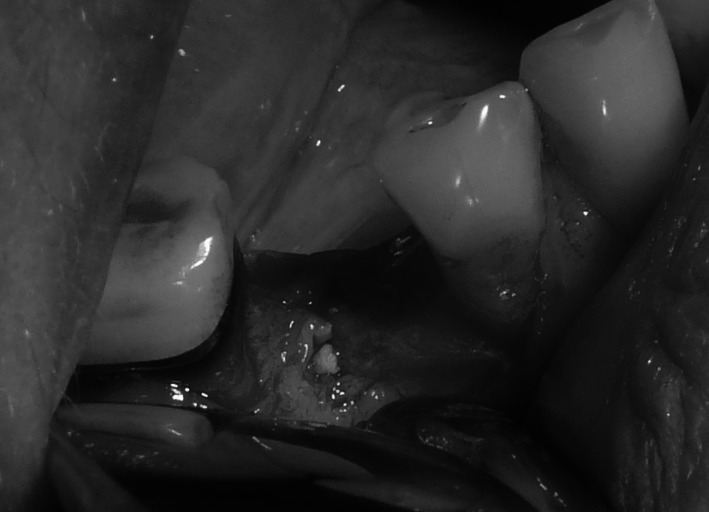
Bone replacement material filling the alveolus

Under local anaesthesia (Xylonor 4 %) trapezoidal incision of the mucous membrane was made in a manner which preserved interdental papillae. Then the mucoperiosteal flap was separated. Intraoral examination revealed a 2 mm defect of the outer bone lamella as well as an alveolar-shaped indentation in the bone filled with bone-replacement material (Fig. 5). Later the material was curetted, which provided bone bed of size exceeding the size of the extracted tooth root for the intraosseous implant with no signs of inflammation. With drills from the implantation box adequate size was obtained and BIOMET 3I implant was embedded. The missing part of the alveolar process was restored with radiation-sterilised (35 kGy), frozen, morselized bone allograft from the Tissue Bank. By cutting the periosteum a flap was mobilised to cover the surgical site. The wound was closed with sutures, which were removed 2 weeks after the procedure was performed; normal healing of the wound margins was found.

**Fig. 6:**
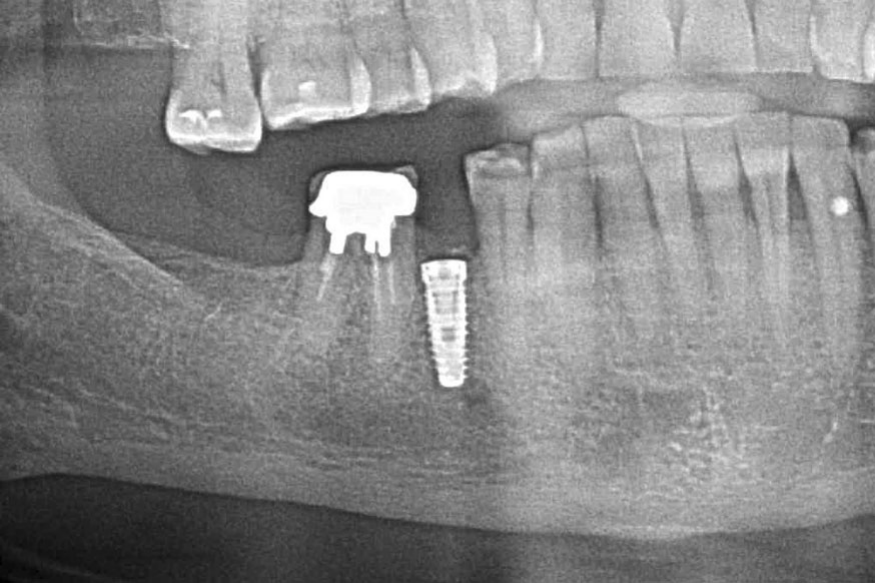
Condition after 6 months of grafting

Following 6 months required for graft reorganisation and integration of the implant with the bone the implant was uncovered and control OPG was taken (Fig. 6). Two weeks later an aesthetic porcelain crown was cemented, which completed implant-prosthetic treatment of this patient.

### Case 3

**Fig. 7:**
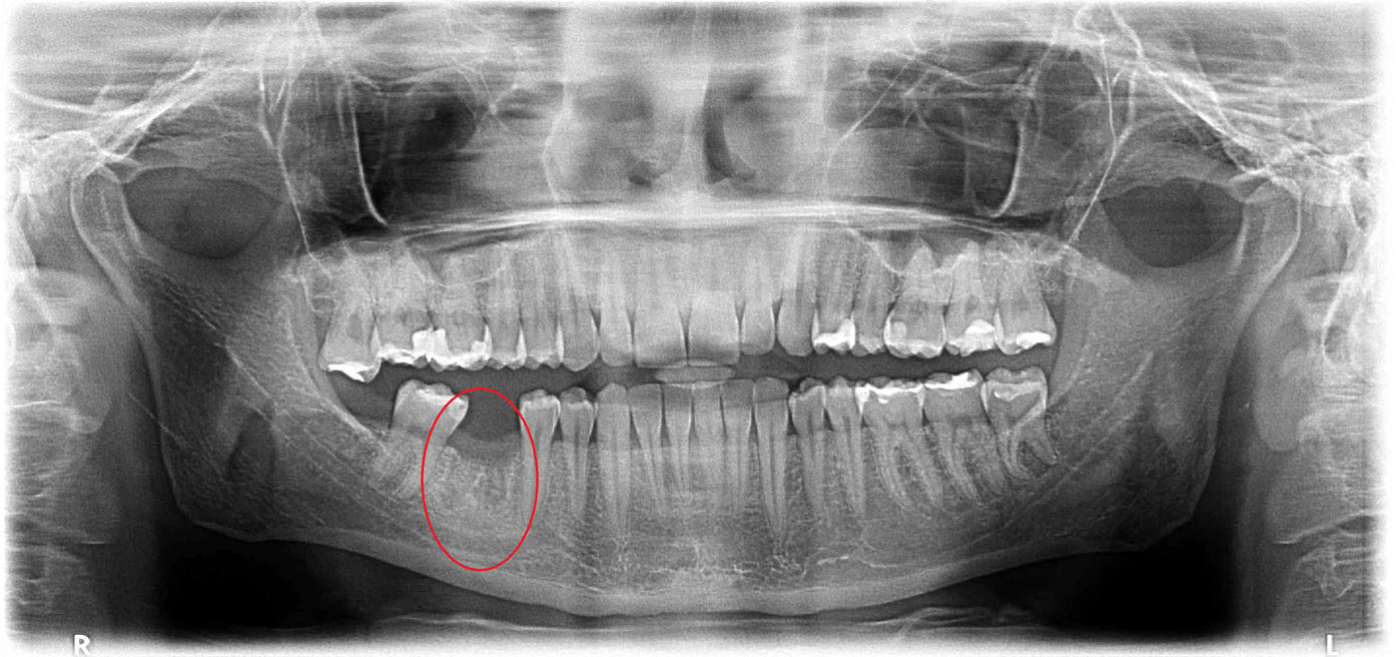
Irregular osteolytic focus of the shape resembling the roots of the extracted tooth 46

Patient A. O. aged 35 reported to restore the missing tooth 46. According to the patient 3 months before due to pain complaints and inflammation the mandibular first right molar was extracted and at the same time the alveolus was filled with bone-replacement material. The patient did not report any pain complaints and condition of the gum did not indicate chronic inflammation. The orthopantomogram revealed irregular osteolytic focus, the shape of which corresponded to the root of the extracted tooth 46 (Fig. 7).

Following thorough intraoral examination and analysis of X-ray images the patient was referred to intraosseous implantation. Due to a bone defect found in the X-ray implantation with simultaneous restoration of the defect in the bone of the alveolar process was suggested.

**Fig. 8:**
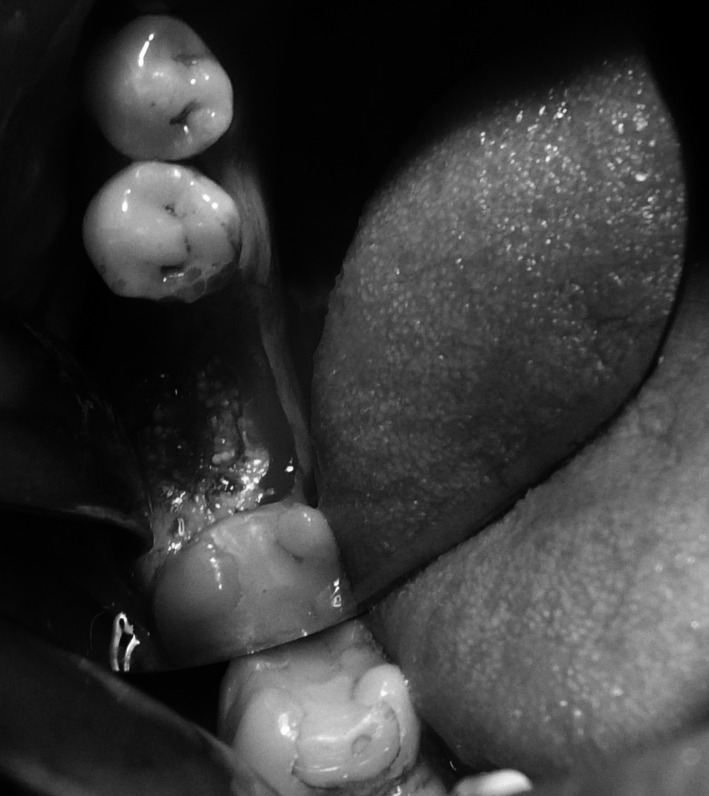
Bone replacement material not integrated with the bone

**Fig. 9:**
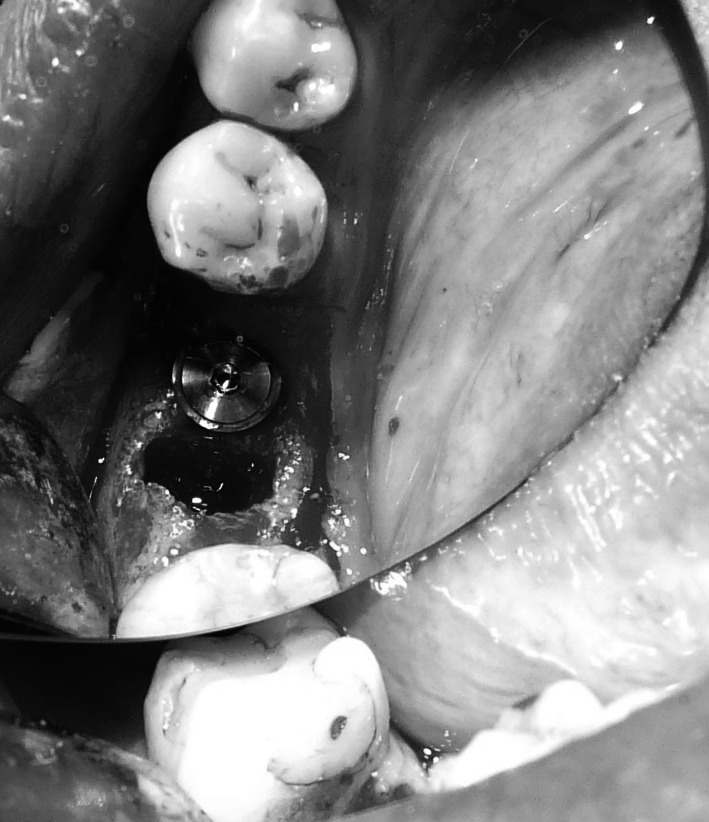
Implant embedded within the area of the mesial root of tooth 46

Under local anaesthesia (Xylonor Forte 4 %) trapezoidal incision of the mucous membrane was made preserving interdental papillae. Then the mucoperiosteal flap was separated. The intraoral examination revealed soft, granulation tissue filling alveoli after the extraction (Fig. 8). The material was curetted out. The remaining part of the patient’s own bone was found to manifest no signs of inflammation and in view of the funnel-like shape of the defect fresh frozen undecalcified radiation-sterilised allogeneic granulate from the tissue bank was chosen to be used, similar to the graft used in the treatment of a patient described in case 2. The implantation box was used to prepare an adequate bone bed at the site of the mesial root and the intraosseous implant was embedded (Fig. 9). The missing bone in the distal root as well as the defect around the neck of the embedded implant were restored with allogeneic bone granulate prepared as in case 2. The flap was mobilised and the wound was sutured.

**Fig. 10:**
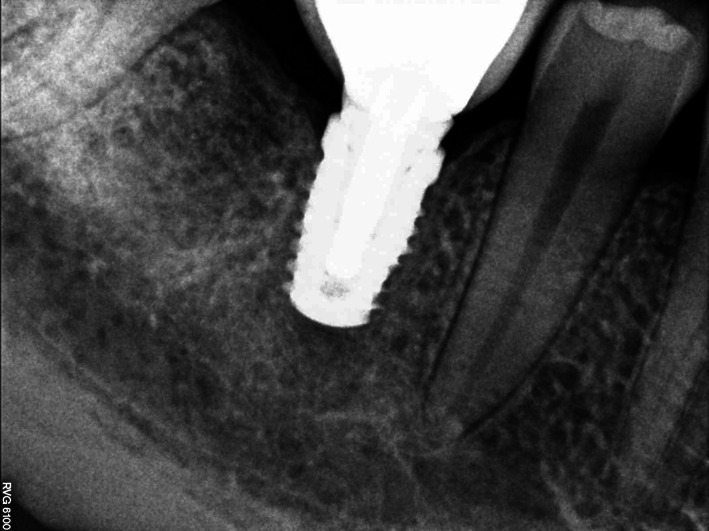
Implant integrated with the bone; visible normal trabecular meshwork at the site of the distal root after 2.5 years of grafting

Three months after the procedure was done, while the implant was being uncovered, normal secondary stabilisation was found as well as optimal height of the alveolar process surrounding the implant. Positive treatment outcome was confirmed with a follow-up X-ray imaging after 2 and a half years (Fig. 10). The result of treatment allowed permanent and aesthetic restoration of the dental defect with a porcelain crown.

### Case 4

**Fig. 11:**
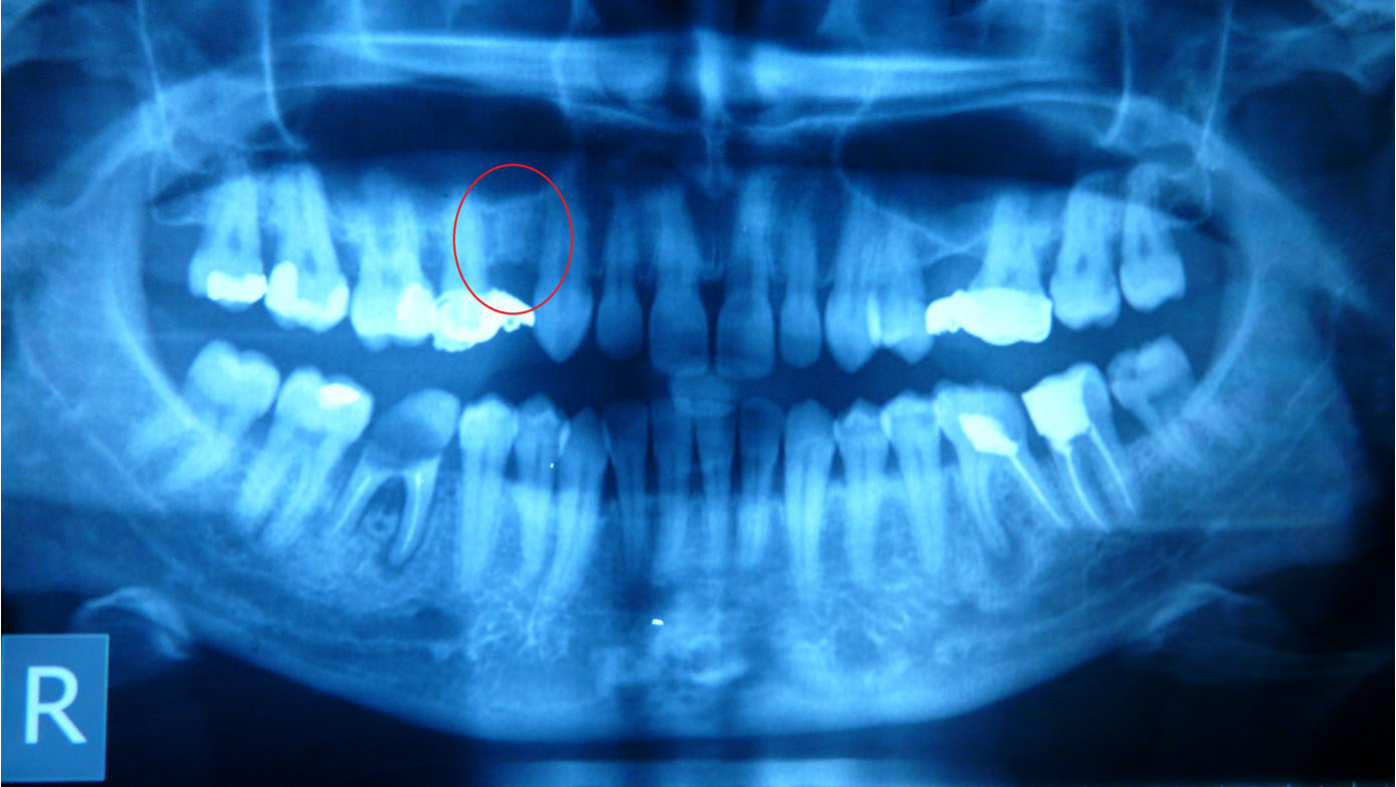
Foreign matter within the area of tooth 14

Patient A. S. aged 32 reported periodically occurring inflammation within the area of tooth 14. The condition was observed following extraction of the right, maxillary first premolar, which was performed over a year before. According to the patient directly after the extraction, bone augmentation with alloplastic material was performed in order to maintain the width of the alveolar process. After the gum healed a cantilever bridge supported by tooth 15. Orthopantomogram performed on the day of examination revealed foreign matter within the area of the extracted root of tooth 14 (Fig. 11).

Based on a physical and radiological examinations dental treatment plan was drawn up. The decision was made to remove the bone replacement material and at the same time to perform implantation within the area of tooth 14. Previously trimmed tooth 15 was qualified for prosthetic restoration with a post and all-porcelain crown.

**Fig. 12:**
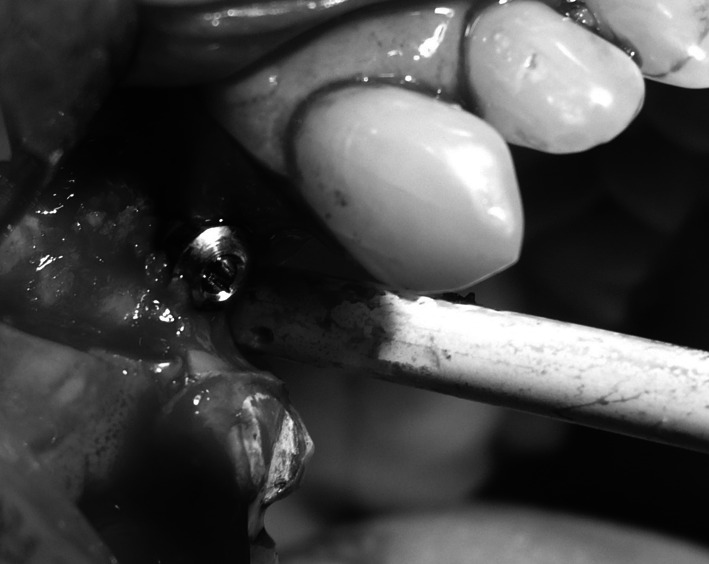
Dental implant embedded in the bone

**Fig. 13:**
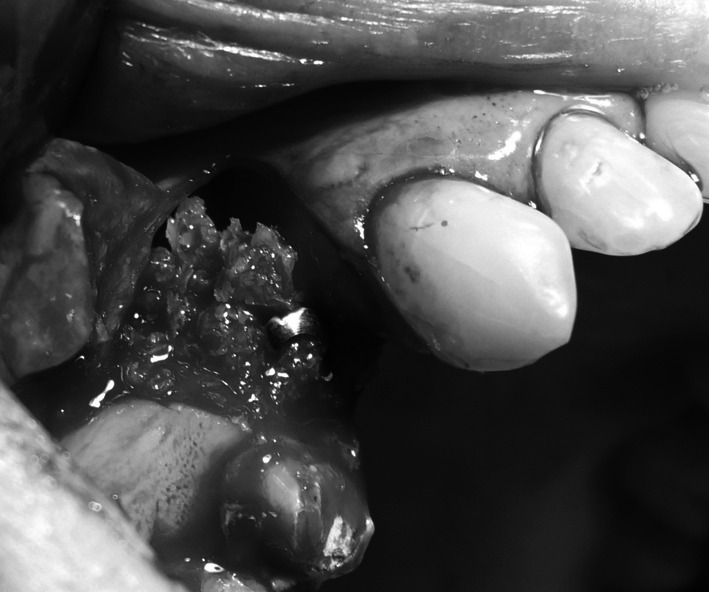
Bone defect around the implant neck covered with morselized bone allograft

Under local anaesthesia (Xylonor Forte 4 %) a trapezoidal incision of the mucous membrane was made, preserving interdental papillae. Then the mucoperiosteal flap was separated, which revealed soft, granulation tissue and granulate material. The material was curetted out leaving the patient’s own bone with no signs of inflammation. The bone defect was trimmed with drills from an implantation box. A bone bed was thus obtained for the implant embedment. Due to the chronic inflammatory condition within the surgical site existing for a year, the implant neck was not correctly submerged in the bone tissue of the process (Fig. 12). The situation required restoration of the bone defect of the process with allogeneic granulate from the tissue bank as previously described in case 2 and 3 (Fig. 13). By cutting the periosteum, the flap was mobilised and the wound was sutured. The sutures were removed at the first follow-up visit after 2 weeks. At the next follow-up visits, 1 and 3 months after the procedure, no relapse of inflammation was found and the mucous membrane around the implant was smooth and shiny.

**Fig. 14:**
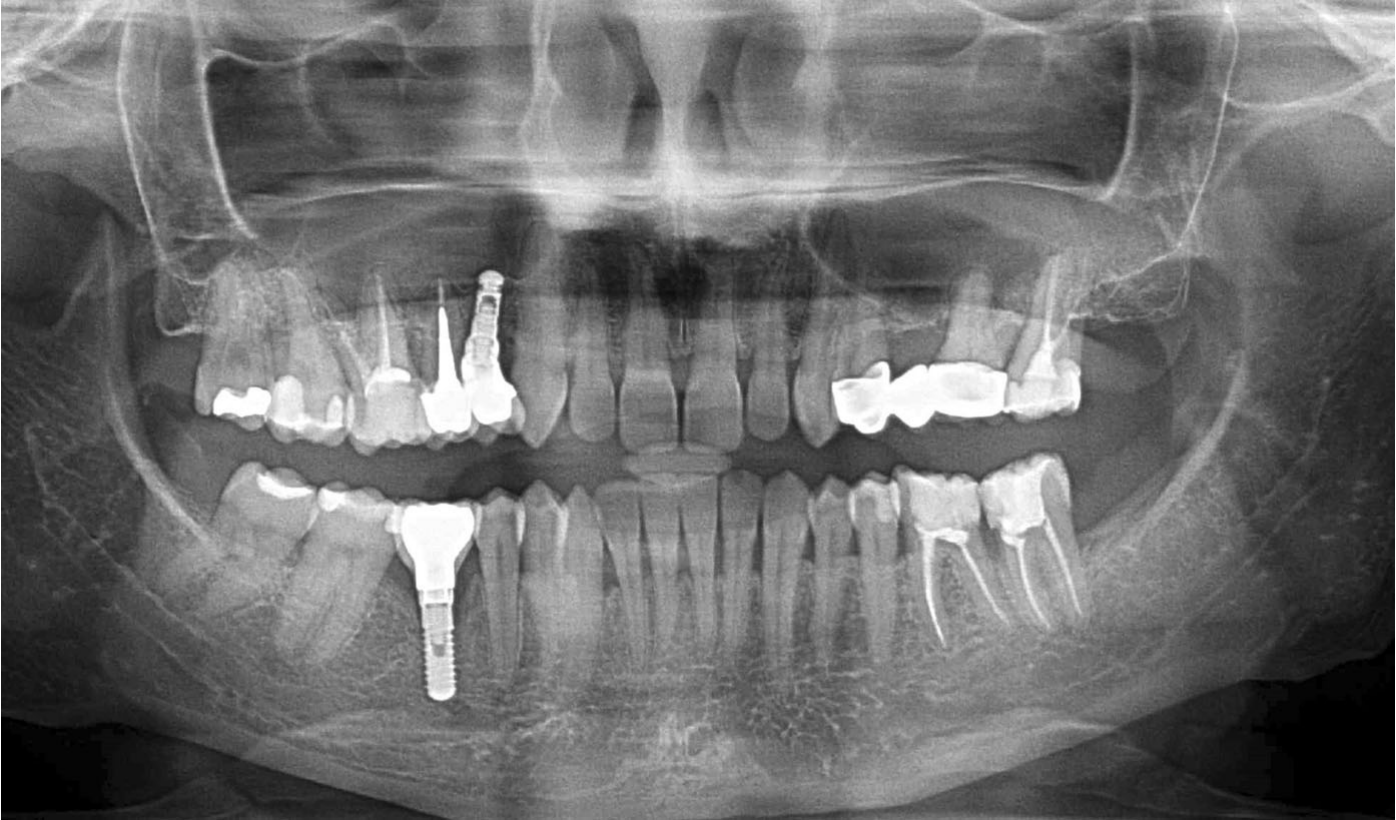
Stable treatment outcome after 6 years of grafting

Following 6 months required for reorganisation of the graft and integration of the implant with the bone, the intraosseous implant was uncovered. Normal integration with the bone as well as optimal level of the alveolar process allowed placement of aesthetic crowns on the root of tooth 15 as well as supported by the implant within the area of tooth 14. Radiological follow-up after 6 years of grafting confirmed the efficacy of the second procedure performed with allogeneic material, normal bone structure, and no signs of inflammation around the implant (Fig. 14).

## Results

During the 2-year follow-up no implant was lost in any of the cases. The high level of function and aesthetics of the prosthetic restorations was maintained as assessed by the patients. Stability of implant-supported porcelain crowns as well as stability of the implants (BIOMET 3I) did not raise any concerns.

## Discussion

Many authors, including authors of this article, believe that autografts are the best material used for reconstruction of alveolar processes. This so called golden standard results from the lack of immunological reaction between the donor and recipient materials. Autograft contains live cells necessary for the process of bone formation, hence presents osteoinductive properties. Limitations of this method include longer duration of the procedure with two surgical sites, where the donor site is weakened for some time. This is associated with higher incidence of post-operational complications and exposing the patient to a longer recovery period (Singh et al. 2009; Silva et al. 2006). Additional intraoral location does not always provide sufficient amount of bone tissue, which limits the use of the method. It does not change the fact that autografts undergo fastest reorganisation and the share of successful procedures is the highest.

The other bone-replacement materials available on the market present a similar, approximately 90–95 % efficacy in procedures of widening the bone of maxillary and mandibular alveolar processes. Their reorganisation lasts longer but numerous studies prove that during long-term observation there is no significant influence of the type of material used on osteointegration, life span of the implant in the site of embedment or the level of bone atrophy around the implant (Jensen and Terheyden 2009; von Arx et al. 2001).

One of the most common mistakes made by inexperienced operators is inadequate selection of the material texture in relation to the type of the atrophied bone base. Another problem is incorrect selection of the material type in view of its properties in regard of conditions necessary for implant integration. It leads to partial reorganisation of the used material or complete lack of union of the material with the patient’s tissue.

For restoration of vertical atrophy bone blocks are the best choice. They are horizontally fixed to the bone of the alveolar process (Khojasteh et al. 2012). Bone atrophy, both, in vertical and horizontal dimensions requires restoration with an adequately prepared, L-shaped bone block. In those cases bone-replacement material with membranes is contraindicated as this form is not rigid enough and does not provide satisfactory scaffold for the newly formed bone of the process. Additionally, it is impossible to fix it firmly, hence small parts of the material will move against the base during mastication in contrast to a block firmly fixed to the recipient site with two screws. As many clinicists state, a concentrate of osteoblasts obtained from the patient’s centrifuged blood (PRF membranes) increases efficacy and reduces the time of implant reorganisation considerably, which is associated with decreased risk of procedure failure (Esposito et al. 2006).

Ground bone replacement material, on the other hand, is recommended to fill the post-extraction alveoli in order to preserve the process against horizontal atrophy (Wang and Tsao 2007). However, the use of non-resorbable granulate may not produce the expected effect, because its reorganisation and replacement with newly formed osseous tissue is not possible, as in case 2. This process may proceed with inflammation (case 4) or with no symptoms as in patients presented in the second and third case report. The granulate is also used in procedures like the sinus lift, where the type of the material seems to be of less significance (Hallman et al. 2002; Aghaloo and Moy 2007).

The authors would also like to draw your attention to problems related to the urge to maximally reduce the duration of alveolar process restoration and to perform implantation at the same time in case of inflammation within the surgical site (Lindeboom et al. 2006). Bacteria and inflammatory cells to a large extent limit regenerative, reparative, and reconstructive abilities of the body as they reduce the inflow of adequate cells. It results in rejection of the graft, exacerbates resorption of the bone as well as inflammation within the alveolar process, which prevents long-term implant restoration (Malo et al. 2012). Often repeated augmentation is necessary, which creates additional challenge for the operator and involves a higher risk of failure (Ortega-Martínez et al. 2012).

Factors that guarantee a success of this type of procedures include both, adequate choice of the type and texture of bone replacement material, its resorbability, the method used as well as the existing condition of the recipient site, the patient’s general condition, and the nature of the bone defect (Khan et al. 2005).

## References

[CR1] Aghaloo TL, Moy PK (2007). Which hard tissue augmentation techniques are the most successful in furnishing bony support for implant placement?. Int J Oral Maxillofac Implants.

[CR2] Bauer TW, Muschler GF (2000). Bone graft materials. An overview of the basic science. Clin Orthop Relat Res.

[CR3] Boyan BD, Ranly DM, Schwartz Z (2006). Use of growth factors to modify osteoinductivity of demineralized bone allografts: lessons for tissue engineering of bone. Dent Clin North Am.

[CR4] Esposito M, Grusovin MG, Coulthard P, Worthington HV (2006). The efficacy of various bone augmentation procedures for dental implants: a Cochrane systematic review of randomized controlled clinical trials. Int J Oral Maxillofac Implants.

[CR5] Hallman M, Sennerby L, Lundgren S (2002). A clinical and histologic evaluation of implant integration in the posterior maxilla after sinus floor augmentation with autogenous bone, bovine hydroxyapatite, or a 20:80 mixture. Int J Oral Maxillofac Implants.

[CR6] Holmquist P, Dasmah A, Sennerby L, Hallman M (2008). A new technique for reconstruction of the atrophied narrow alveolar crest in the maxilla using morselized impacted bone allograft and later placement of dental implants. Clin Implant Dent Relat Res.

[CR7] Jensen SS, Terheyden H (2009). Bone augmentation procedures in localized defects in the alveolar ridge: clinical results with different bone grafts and bone-substitute materials. Int J Oral Maxillofac Implants.

[CR8] Khan SN, Cammisa FP, Sandhu HS, Diwan AD, Girardi FP, Lane JM (2005). The biology of bone grafting. J Am Acad Orthop Surg.

[CR9] Khojasteh A, Behnia H, Shayesteh YS, Morad G, Alikhasi M (2012). Localized bone augmentation with cortical bone blocks tented over different particulate bone substitutes: a retrospective study. Int J Oral Maxillofac Implants.

[CR10] Lindeboom JA, Tjiook Y, Kroon FH (2006). Immediate placement of implants in peri-apical infected sites: a prospective randomized study in 50 patients. Oral Surg Oral Med Oral Pathol Oral Radiol Endod.

[CR11] Malo P, Nobre Mde A, Lopes A (2012). Immediate rehabilitation of completely edentulous arches with a four-implant prosthesis concept in difficult conditions: an open cohort study with a mean follow-up of 2 years. Int J Oral Maxillofac Implants.

[CR12] Ortega-Martínez J, Pérez-Pascual T, Mareque-Bueno S, Hernández-Alfaro F, Ferrés-Padró E (2012). Immediate implants following tooth extraction. A systematic review. Med Oral Patol Oral Cir Bucal.

[CR13] Silva FM, Cortez AL, Moreira RW, Mazzonetto R (2006). Complications of intraoral donor site for bone grafting prior to implant placement. Implant Dent.

[CR14] Singh JR, Nwosu U, Egol KA (2009). Long-term functional outcome and donor-site morbidity associated with autogenous iliac crest bone grafts utilizing a modified anterior approach. Bull NYU Hosp Joint Dis.

[CR15] von Arx T, Cochran DL, Hermann JS, Schenk RK, Higginbottom FL, Buser D (2001). Lateral ridge augmentation and implant placement: an experimental study evaluating implant osseointegration in different augmentation materials in the canine mandible. Int J Oral Maxillofac Implants.

[CR16] Wang HL, Tsao YP (2007). Mineralized bone allograft-plug socket augmentation: rationale and technique. Implant Dent.

